# Supplementary material for: Microencapsulation and nanowarming enables vitrification cryopreservation of mouse preantral follicles
Source: Nat Commun. 2022 Dec 15;13:7515. doi: 10.1038/s41467-022-34549-2 (PMC9755531; doi:10.1038/s41467-022-34549-2)
Supplement: Supplementary file 1 — Supplementary Information [file 41467_2022_34549_MOESM1_ESM.pdf]

Supplementary Materials for

Microencapsulation and Nano-warming Enables Vitrification Cryopreservation of Mouse Preantral Follicles

Conghui Tian[1], Lingxiao Shen[1], Chenjia Gong[2], Yunxia Cao[3, 4], Qinghua Shi[2], Gang Zhao[1]

[1]Department of Electronic Engineering and Information Science, University of Science and Technology of China, Hefei 230027, China

[2]Division of Reproduction and Genetics, First Affiliated Hospital of USTC, Hefei National Laboratory for Physical Science at Microscale, the CAS Key Laboratory of Innate Immunity and Chronic Disease, School of Basic Medical Sciences, Division of Life Sciences and Medicine, Biomedical Sciences and Health Laboratory of Anhui Province, CAS center for Excellence in Molecular Cell Science, Collaborative Innovation Center of Genetics and Development, University of Science and Technology of China, Hefei 230027, China

[3]Department of Obstetrics and Gynecology, the First Affiliated Hospital of Anhui Medical University, Hefei 230022, China

[4]NHC Key Laboratory of Study on Abnormal Gametes and Reproductive Tract (Anhui Medical University), Anhui Provincial Engineering Research

Center of Biopreservation and Artificial Organs, Hefei 230032, China

Correspondence should be addressed to G.Z. (email: zhaog@ustc.edu.cn) and Q.S. (email: qshi@ustc.edu.cn).

This Supplementary PDF file includes:

Supplementary Figures 1 to 12

Supplementary Movies 1 to 5

Supplementary Table 1

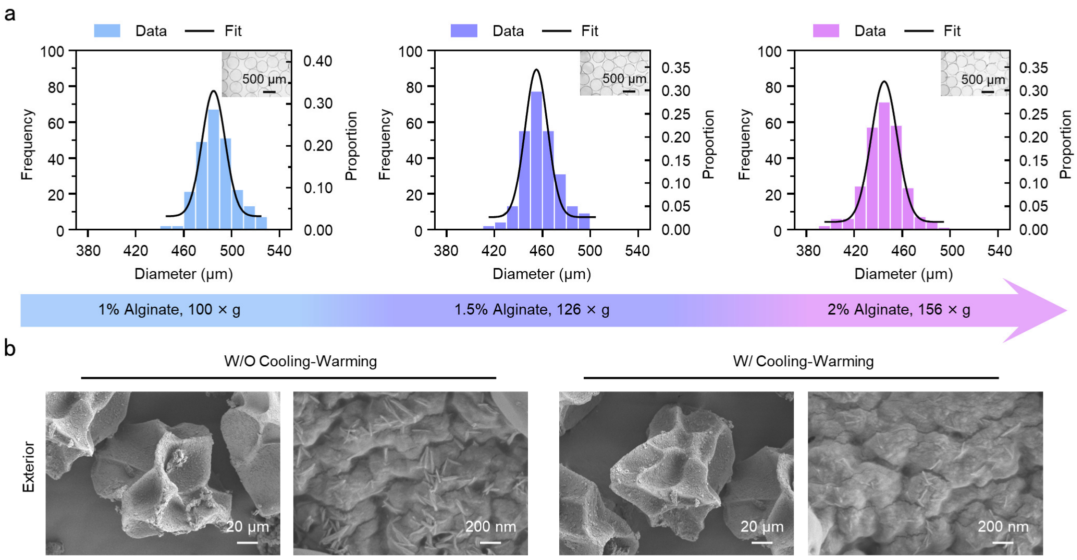

Supplementary Fig. 1 The regulation of PAF microencapsulation and characterization of the hydrogel microcapsules. (a) Morphology and size of alginate hydrogel microcapsules with different concentrations. (b) Scanning electron microscope (SEM) images of 1% (w/v) alginate hydrogel microcapsules before and after freeze-thaw. Each experiment was repeated three times with similar results (b). W/O Cooling-Warming: without cooling-warming. W/ Cooling-Warming: with cooling-warming.

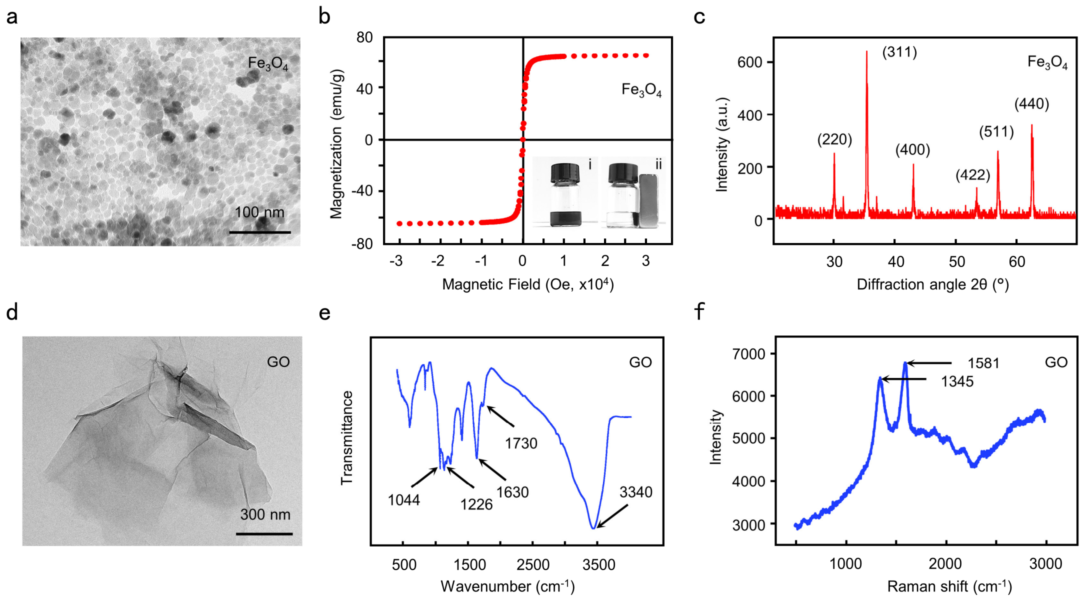

Supplementary Fig. 2 Characterization of Fe_3O_4 and GO NPs. (a) Transmission electron microscope (TEM) image of Fe_3O_4 NPs. Each experiment was repeated three times with similar results. (b) Magnetization curve of Fe_3O_4 nanoparticles (NPs). The inset image shows the dispersion of Fe_3O_4 NPs in DI water. (c) X-Ray diffraction (XRD) pattern of Fe_3O_4 NPs. (d) TEM image of GO NPs. Each experiment was repeated three times with similar results. (e) Fourier transform infrared spectroscopy (FTIR) of GO NPs. (f) Raman spectrum of the GO NPs.

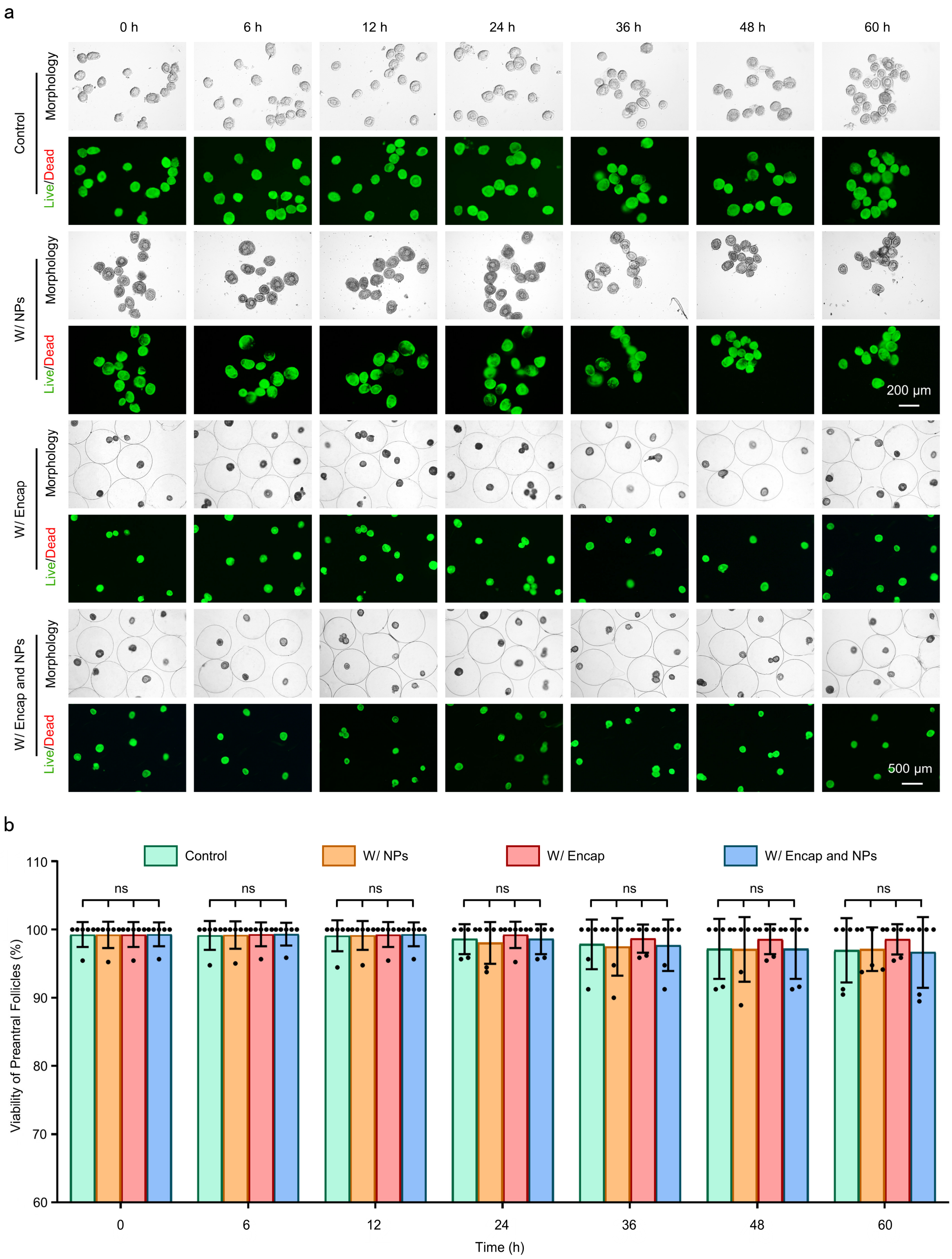

Supplementary Fig. 3 Cytotoxicity of Fe_3O_4 and GO NPs. (a) Fluorescence images of PAFs with (W/) or without (W/O) encapsulation after incubation W/ or W/O NPs for different times (0, 6, 12, 24, 36, 48, 60 h) at 37°C. Each experiment was repeated three times with similar results.

(b) Analysis of PAF survival. Control: fresh PAF; W/ NPs: with NPs (0.3% Fe_3O_4 and 0.03% GO, w/v) but without encapsulation; W/ Encap: with encapsulation; W/ Encap and NPs: with encapsulation and NPs (0.3% Fe_3O_4 and 0.03% GO, w/v). Two-way analysis of variance (ANOVA) and Tukey's post hoc were used for statistical analysis. ns: $p > 0.05$; n = 15-25 for six replicates. n: number of follicles used in each experiment. Data are presented as the mean ± standard deviation (SD) (b).

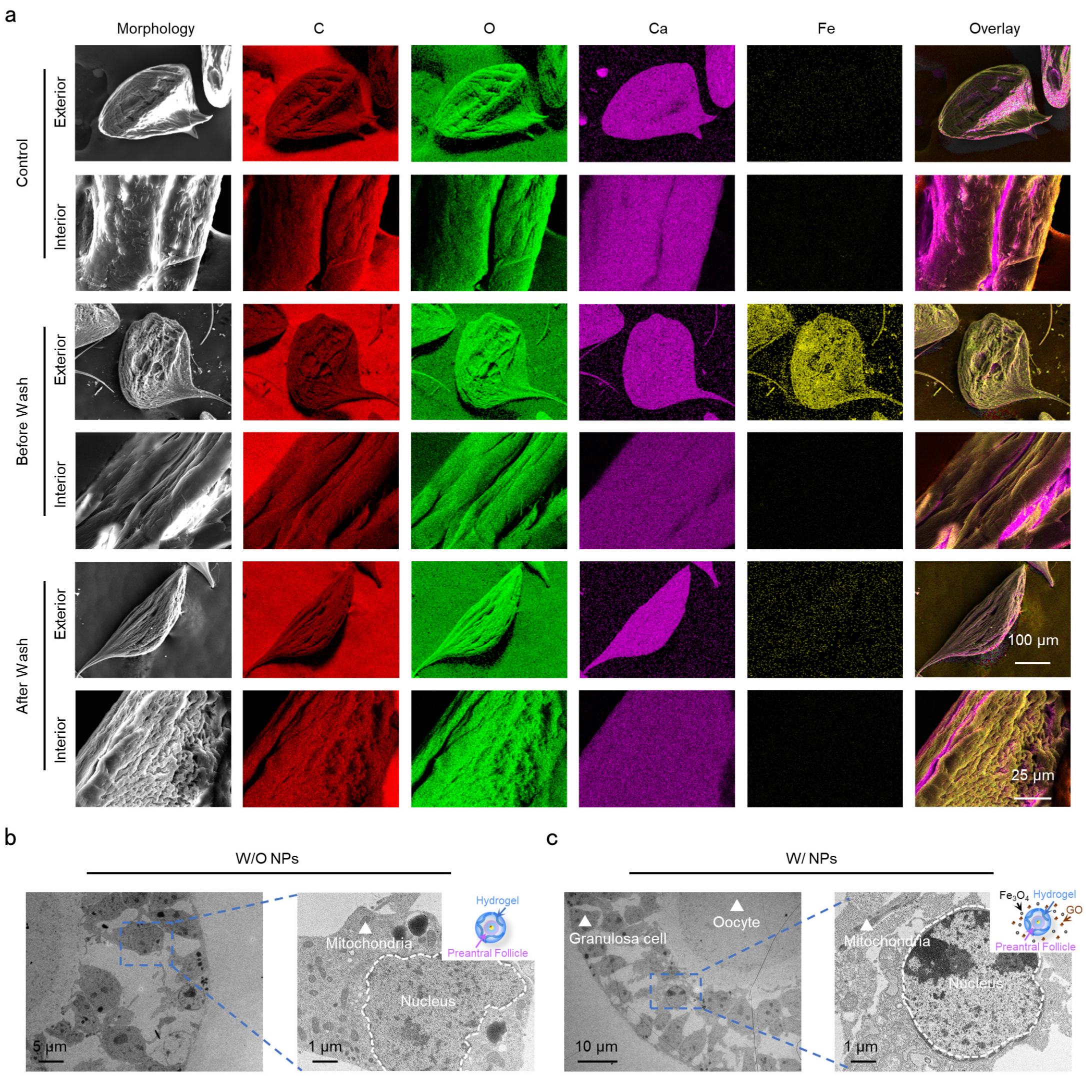

Supplementary Fig. 4 Energy dispersive X-ray spectroscopy (EDS) of the hydrogel microspheres and TEM of PAFs. (a) EDS images of hydrogel microspheres before and after washing. (b) TEM of PAFs encapsulated in 1% alginate hydrogel after incubation W/O NPs. (c) TEM of PAFs encapsulated in 1% alginate hydrogel after incubation W/ NPs. Control: 1% alginate hydrogel microspheres after incubation in DI water. Before Wash: 1% Alginate hydrogel microspheres after incubation in DI water with NPs (0.3% Fe_3O_4 and 0.03% GO, w/v). After Wash: hydrogel

microspheres after removal of NPs in solution by washing. W/O NPs: without NPs. W/ NPs: with NPs (0.3% Fe_3O_4 and 0.03% GO, w/v). Each experiment was repeated three times with similar results (a, b).

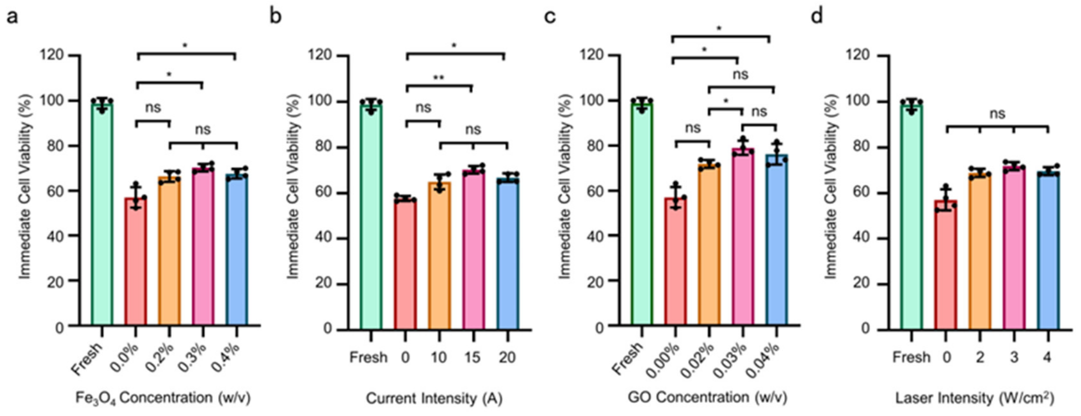

Supplementary Fig. 5 Effect of MIH and LIH on vitrification. (a) Viability of PAFs encapsulated in 1% alginate hydrogel with different concentrations of Fe_3O_4 post-vitrification at a current of 15 A. $P_{0.0\%\text{-}0.3\%} = 0.0298$, $P_{0.0\%\text{-}0.4\%} = 0.0392$. (b) Viability of PAFs encapsulated in 1% alginate with 0.3% Fe_3O_4 after nano-warming with MIH under different current intensity. $P_{0\text{-}15} = 0.0011$, $P_{0\text{-}20} = 0.0184$. (c) Viability of PAFs encapsulated in 1% alginate hydrogel with different concentrations of GO post-vitrification at a laser of 3 W/cm^2. $P_{0.00\%\text{-}0.03\%} = 0.0183$, $P_{0.00\%\text{-}0.04\%} = 0.0326$, $P_{0.02\%\text{-}0.03\%} = 0.0241$. (d) Viability of PAFs encapsulated in 1% alginate with 0.03% GO after nano-warming with LIH under different laser intensity. One-way analysis of variance (ANOVA) and Tukey's post hoc were used for statistical analysis. ns: $p > 0.05$; *: $p < 0.05$; **: $p < 0.01$. n = 15-25 for four replicates. n: number of follicles used in each experiment. Data are presented as the mean ± SD (a, b, c, d).

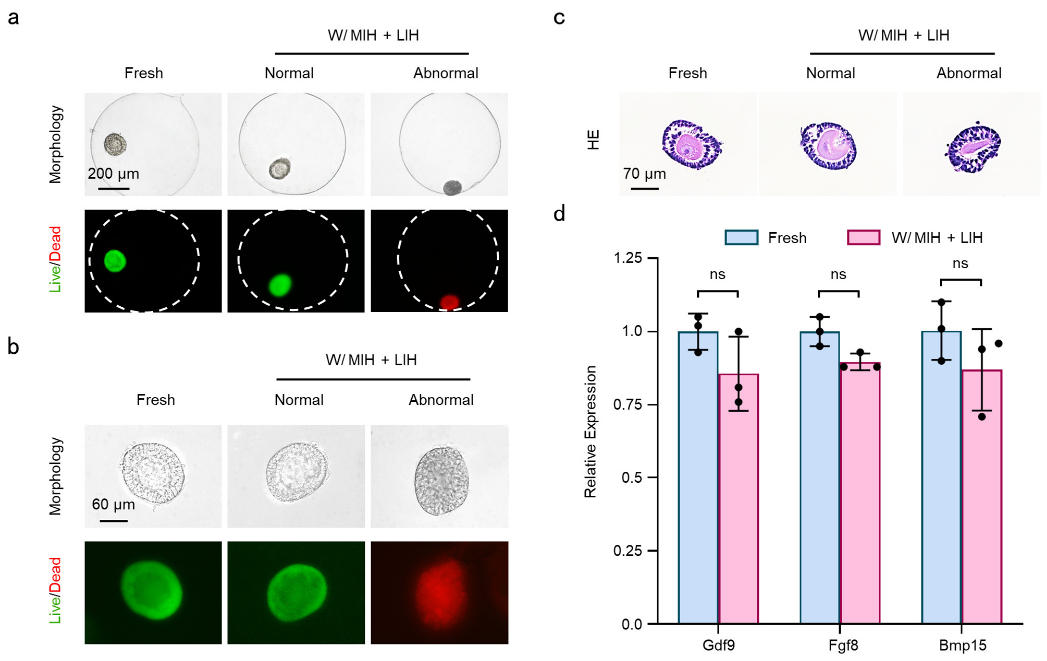

Supplementary Fig. 6 Effect of vitrification and warming with LIH and MIH on the morphology and mRNA expression of PAFs. (a) Representative DIC images and fluorescence images of PAFs encapsulated in hydrogel after warming. (b) Representative DIC images and fluorescence images of PAFs after dissolving the hydrogel. (c) Typical histological images of PAFs after warming. (d) mRNA expression of PAF genes associated with development *in vitro* (Gdf9, Fgf8 and Bmp15). Fresh: PAFs obtained directly from mice. W/ MIH + LIH: PAFs after vitrification and nano-warming with MIH + LIH. Two-way analysis of variance (ANOVA) and Tukey's post hoc were used for statistical analysis. ns: $p > 0.05$; n = 10-20 for three replicates. n: number of follicles used in each experiment. Data are presented as the mean ± SD (d). Each experiment was

repeated three times with similar results (a, b, c).

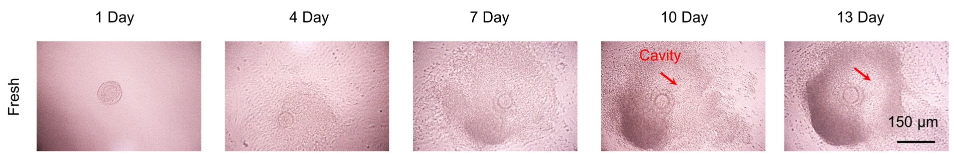

Supplementary Fig. 7 Typical developmental images of fresh PAF without (W/O) encapsulation. Fresh: PAFs obtained directly from mice. Each experiment was repeated three times with similar results.

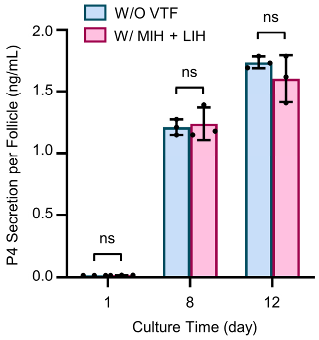

Supplementary Fig. 8 Secretion of progesterone (P4) from growing PAFs. W/O VTF: PAFs were only encapsulated in hydrogel and did not undergo vitrification; W/ MIH + LIH: PAFs encapsulated in hydrogel were warmed with MIH and LIH after vitrification. Two-way analysis of variance (ANOVA) and Tukey's post hoc were used for statistical analysis. ns: $p > 0.05$. n = 10-20 for three replicates. n: number of follicles used in each experiment. Data are presented as the mean ± SD.

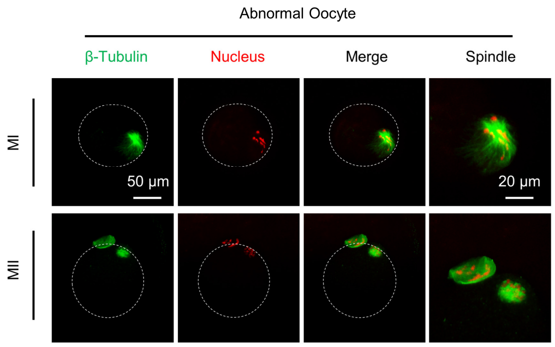

Supplementary Fig. 9 Fluorescence images of abnormal spindles. Each experiment was repeated three times with similar results.

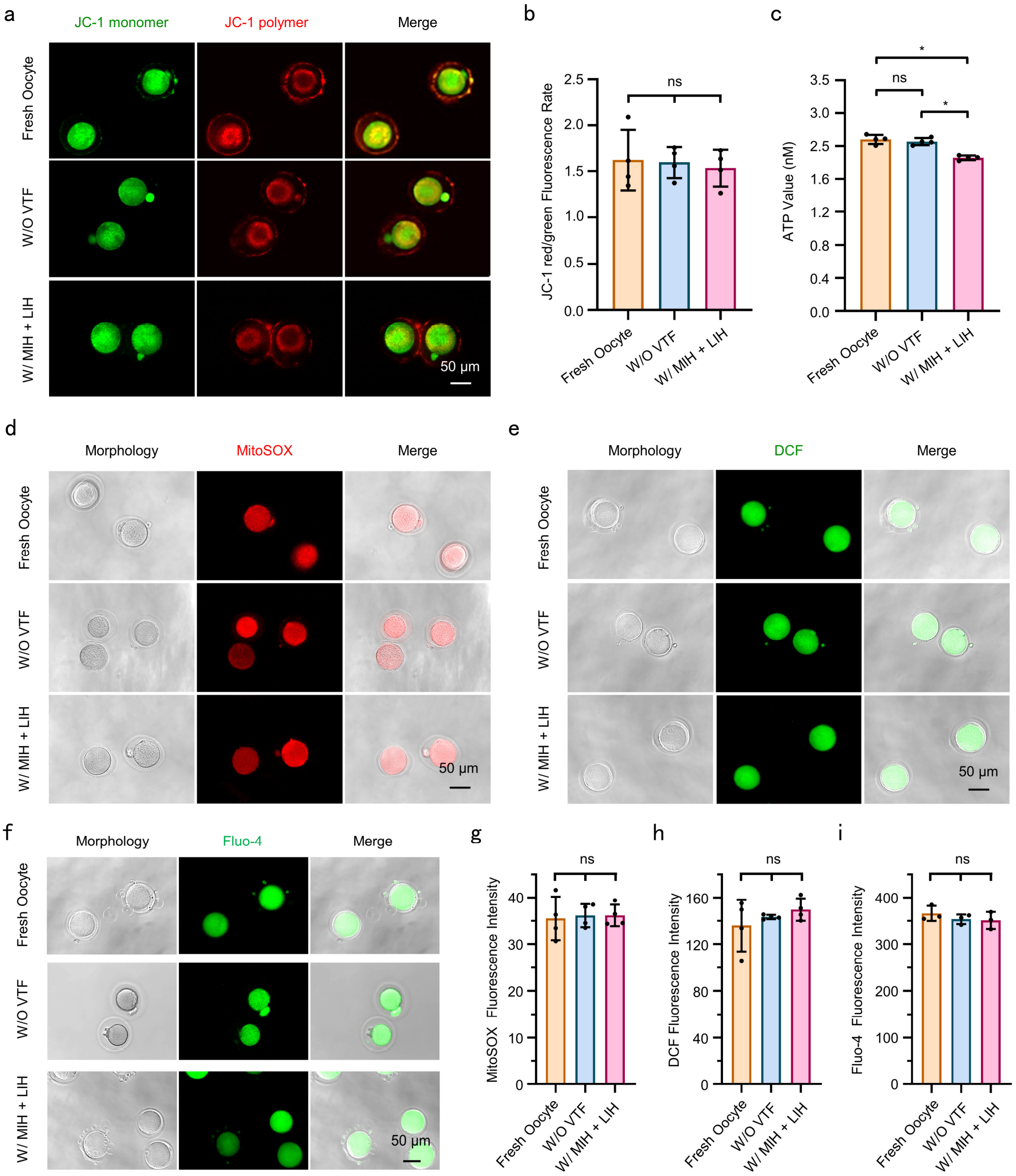

Supplementary Fig. 10 Detection of mitochondrial function of oocytes. (a) The MMP of oocytes was measured by JC-1 staining. (b) Analysis of JC-1 fluorescence staining using a microplate reader. (c) ATP content in oocytes. $P_{\text{Fresh-W/ MIH + LIH}} = 0.0163$, $P_{\text{W/O VTF-W/ MIH + LIH}} = 0.0219$. (d) MitoSOX staining was performed to evaluate the level of MS in oocytes. (e) The level of ROS in oocytes was assessed by DCFH-DA staining. (f)

Fluo-4 staining was performed to assess the levels of calcium in oocytes. (g) Fluorescence intensity of MitoSOX analysis. (h) Fluorescence intensity of DCFH-DA analysis. (i) Fluorescence intensity of Fluo-4 analysis. One-way analysis of variance (ANOVA) and Tukey's post hoc were used for statistical analysis (b, c, h, g, i). ns: $p > 0.05$; *: $p < 0.05$. n = 10-20 for three replicates (b, c, h, i); n = 10-20 for four replicates (g). n: number of follicles used in each experiment. W/O VTF: PAFs were only encapsulated in hydrogel and did not undergo vitrification; W/ MIH + LIH: PAFs encapsulated in hydrogel were warmed with MIH and LIH after vitrification; Fresh oocytes: MII oocytes obtained directly from the ampulla of the oviduct of a mouse. Data are presented as the mean ± SD (b, c, g, h, i). Each experiment was repeated three times with similar results (a, d, e, f).

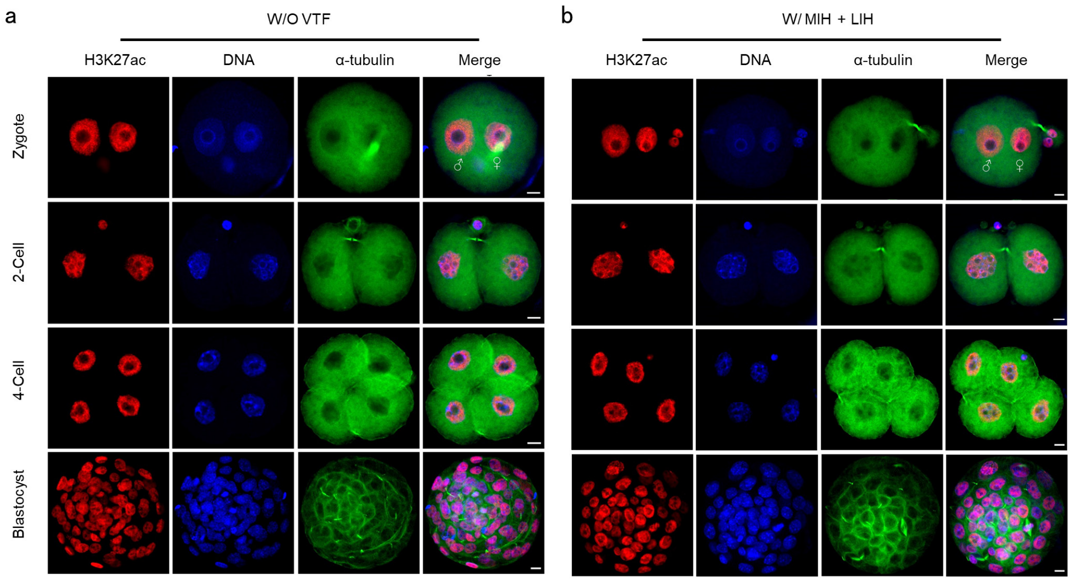

Supplementary Fig. 11 Representative images of H3K27ac in embryos at different stages. (a) Fluorescence images of H3K27ac in embryos at different stages in the W/O VTF group. (b) Fluorescence images of H3K27ac in embryos at different stages in the W/ MIH + LIH group. Scale bar is 10 μm (a and b). W/O VTF: PAFs were only encapsulated in hydrogel and did not undergo vitrification; W/ MIH + LIH: PAFs encapsulated in hydrogel were warmed with MIH and LIH after vitrification. Each experiment was repeated three times with similar results.

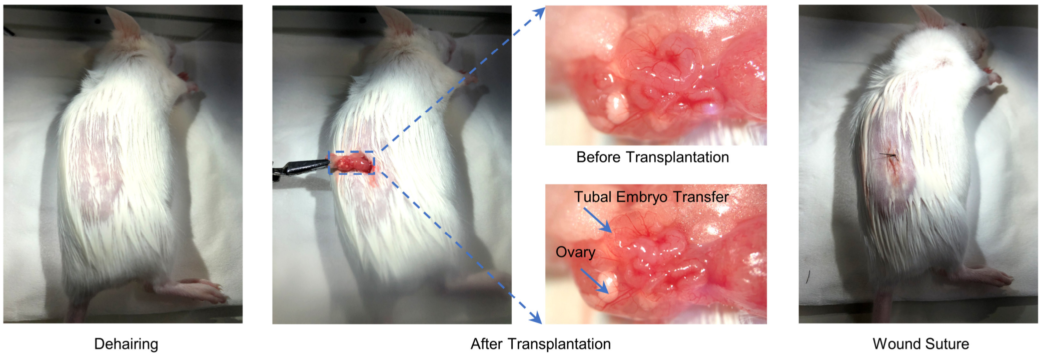

Supplementary Fig. 12 A demonstration of the surgical process in which two-cell embryos are transplanted into surrogate mice.

Supplementary Table 1 Primer sequences of Gdf9, Bmp15 and Fgf8.

Gene	Primer
Gdf9	Forward primer: CTTCAGCCCCTAGTGACCTC<br>Reverse primer: GAGGCATGCTAAACACTCCG
Bmp15	Forward primer: GAAGCTCTGGAATCGCAAGG<br>Reverse primer: CCCTGAACTCTGTCATCGGT
Fgf8	Forward primer: GGAAGCAGAGTCCGAGTTC<br>Reverse primer: GCCGTTGCTCTTGGCAATTA
