# Supplementary material for: Microencapsulation and nanowarming enables vitrification cryopreservation of mouse preantral follicles
Source: Nat Commun. 2022 Dec 15;13:7515. doi: 10.1038/s41467-022-34549-2 (PMC9755531; doi:10.1038/s41467-022-34549-2)
Supplement: Supplementary file 3 — Description of Additional Supplementary Files [file 41467_2022_34549_MOESM3_ESM.pdf]

Description of Additional Supplementary Files

File Name: Supplementary Movie 1
Description: The microencapsulation process of PAFs *via* a centrifugal microfluidic device.

File Name: Supplementary Movie 2
Description: The process from the beginning of the growth of ice crystals to complete melting under warming of CPA in a PS in a 37°C water bath.

File Name: Supplementary Movie 3
Description: The process from the beginning of the growth of ice crystals to complete melting under warming of CPA in PS with MIH (15 A) in a 37°C water bath.

File Name: Supplementary Movie 4
Description: The process from the beginning of the growth of ice crystals to complete melting under warming of CPA in PS with LIH (3 W/cm2) in a 37°C water bath.

File Name: Supplementary Movie 5
Description: The process from the beginning of the growth of ice crystals to complete melting under warming of CPA in PS with MIH and LIH (15 A, 3 W/cm2) in a 37°C water bath.
