# Supplementary material for: Microencapsulation and nanowarming enables vitrification cryopreservation of mouse preantral follicles
Source: Nat Commun. 2022 Dec 15;13:7515. doi: 10.1038/s41467-022-34549-2 (PMC9755531; doi:10.1038/s41467-022-34549-2)
Supplement: Supplementary file 9 — Reporting Summary [file 41467_2022_34549_MOESM9_ESM.pdf]

nature portfolio

Corresponding author(s): Qinghua Shi, Gang Zhao

Last updated by author(s): Oct 10, 2022

Reporting Summary

Nature Portfolio wishes to improve the reproducibility of the work that we publish. This form provides structure for consistency and transparency in reporting. For further information on Nature Portfolio policies, see our Editorial Policies and the Editorial Policy Checklist.

Statistics

For all statistical analyses, confirm that the following items are present in the figure legend, table legend, main text, or Methods section.

n/a	Confirmed	
☐	☒	The exact sample size (*n*) for each experimental group/condition, given as a discrete number and unit of measurement
☐	☒	A statement on whether measurements were taken from distinct samples or whether the same sample was measured repeatedly
☐	☒	The statistical test(s) used AND whether they are one- or two-sided<br>*Only common tests should be described solely by name; describe more complex techniques in the Methods section.*
☒	☐	A description of all covariates tested
☐	☒	A description of any assumptions or corrections, such as tests of normality and adjustment for multiple comparisons
☐	☒	A full description of the statistical parameters including central tendency (e.g. means) or other basic estimates (e.g. regression coefficient) AND variation (e.g. standard deviation) or associated estimates of uncertainty (e.g. confidence intervals)
☐	☒	For null hypothesis testing, the test statistic (e.g. *F*, *t*, *r*) with confidence intervals, effect sizes, degrees of freedom and *P* value noted<br>*Give P values as exact values whenever suitable.*
☒	☐	For Bayesian analysis, information on the choice of priors and Markov chain Monte Carlo settings
☒	☐	For hierarchical and complex designs, identification of the appropriate level for tests and full reporting of outcomes
☒	☐	Estimates of effect sizes (e.g. Cohen's *d*, Pearson's *r*), indicating how they were calculated

Our web collection on statistics for biologists contains articles on many of the points above.

Software and code

Policy information about availability of computer code

Data collection	NIS Elements software (Nikon) was used to collect the data
Data analysis	Graphpad prism 8, ImageJ 1.52a, Image-Pro Plus 6.0 were used to analyze the data

For manuscripts utilizing custom algorithms or software that are central to the research but not yet described in published literature, software must be made available to editors and reviewers. We strongly encourage code deposition in a community repository (e.g. GitHub). See the Nature Portfolio guidelines for submitting code & software for further information.

Data

Policy information about availability of data

All manuscripts must include a data availability statement. This statement should provide the following information, where applicable:

- Accession codes, unique identifiers, or web links for publicly available datasets
- A description of any restrictions on data availability
- For clinical datasets or third party data, please ensure that the statement adheres to our policy

The authors declare that all data supporting the findings of this study are available within the paper and its supplementary information files. Source data are provided with this paper. Data availability statement has been included in the manuscript.

Human research participants

Policy information about studies involving human research participants and Sex and Gender in Research.

Reporting on sex and gender	Not applicable
Population characteristics	Not applicable
Recruitment	Not applicable
Ethics oversight	Not applicable

Note that full information on the approval of the study protocol must also be provided in the manuscript.

Field-specific reporting

Please select the one below that is the best fit for your research. If you are not sure, read the appropriate sections before making your selection.

☒ Life sciences ☐ Behavioural & social sciences ☐ Ecological, evolutionary & environmental sciences

For a reference copy of the document with all sections, see nature.com/documents/nr-reporting-summary-flat.pdf

Life sciences study design

All studies must disclose on these points even when the disclosure is negative.

Sample size	No specific statistical methods were used to predetermine the sample size. According to the experience in previous related studies in this field (Monica M. Laronda et al, Nature Communications 2017; Li Zhan et al, Nature Communications 2021; Yan Zhang et al, Nature Communications 2021; Wenqi Hu et al,Nature Communications 2022) to choose an adequate pool for reporter assay, microscopy, qTR-PCR, and mouse experiments. For each experiment, the samples used were indicated in the figures or legends. These numbers of samples were sufficient to perform a confident data analysis.
Data exclusions	No data was excluded from our analyses.
Replication	All data presented are representative of at least three independent experiments as indicated in the figure legends, and replications were successful.
Randomization	Follicles used in experiments were collected randomly from 3-week-old KM female mice. Samples were allocated into experimental groups randomly.
Blinding	No blinding was used for the data collection or analysis.

Reporting for specific materials, systems and methods

We require information from authors about some types of materials, experimental systems and methods used in many studies. Here, indicate whether each material, system or method listed is relevant to your study. If you are not sure if a list item applies to your research, read the appropriate section before selecting a response.

Materials & experimental systems

n/a	Involved in the study	
☐	☒	Antibodies
☒	☐	Eukaryotic cell lines
☒	☐	Palaeontology and archaeology
☐	☒	Animals and other organisms
☒	☐	Clinical data
☒	☐	Dual use research of concern

Methods

n/a	Involved in the study	
☒	☐	ChIP-seq
☒	☐	Flow cytometry
☒	☐	MRI-based neuroimaging

Antibodies

Antibodies used	Anti-β-tubulin antibody (SAB4200715, Sigma-Aldrich) (1:500 dilution)<br>Alexafluor 488 rabbit anti-mouse IgG (H + L) secondary antibody (A-11059, Invitrogen) (1:500 dilution)

	Anti-α-tubulin-FITC, F2168, Sigma-Aldrich (1:500 dilution)<br>Anti-H3K9me3 antibody (A2360, ABclonal, 1:100 dilution)<br>Anti-H3K4me3 antibody (ab8580, Abcam, 1:100 dilution)<br>Anti-H3K27ac antibody (8173, Cell Signaling Technology, 1:100 dilution)<br>Donkey anti-rabbit IgG (H + L) highly cross-adsorbed secondary antibody Alexa Fluor 555 (A-11059, Invitrogen) (1:500 dilution)
Validation	Anti-β-tubulin antibody: reactivity validated by Sigma-Aldrich; validated in immunocytochemistry (Sigma-Aldrich Sci website, under product specification);<br>Anti-α-tubulin-FITC: reactivity validated by Sigma-Aldrich; validated in immunocytochemistry (Sigma-Aldrich Sci website, under product specification);<br>lexafluor 488 rabbit anti-mouse IgG (H + L) secondary antibody: reactivity validated by Invitrogen; validated in immunocytochemistry (ThermoFisher Sci website, under product specification);<br>Anti-H3K9me3 antibody: reactivity validated by ABclonal; validated in immunocytochemistry (ABclonal Sci website, under product specification);<br>Anti-H3K4me3 antibody: reactivity validated by Abcam; validated in immunocytochemistry (Abcam Sci website, under product specification);<br>Anti-H3K27ac antibody: reactivity validated by Cell Signaling Technology; validated in immunocytochemistry (Cell Signaling Technology Sci website, under product specification);<br>Donkey anti-rabbit IgG (H + L) highly cross-adsorbed secondary antibody Alexa Fluor 555: reactivity validated by Invitrogen; validated in immunocytochemistry (ThermoFisher Sci website, under product specification).

Animals and other research organisms

Policy information about studies involving animals; ARRIVE guidelines recommended for reporting animal research, and Sex and Gender in Research

Laboratory animals	Three-week-old female and male KM mice were used in (Vital River, China).
Wild animals	No wild animals were used.
Reporting on sex	This finding just apply to female.
Field-collected samples	The study did not involve samples collected from field.
Ethics oversight	All operations and research were performed in accordance with the protocol of the animal ethics committee of the University of Science and Technology of China.

Note that full information on the approval of the study protocol must also be provided in the manuscript.
